# Epidermal growth factor gene is a newly identified candidate gene for gout

**DOI:** 10.1038/srep31082

**Published:** 2016-08-10

**Authors:** Lin Han, Chunwei Cao, Zhaotong Jia, Shiguo Liu, Zhen Liu, Ruosai Xin, Can Wang, Xinde Li, Wei Ren, Xuefeng Wang, Changgui Li

**Affiliations:** 1 Shandong Gout Clinical Medical Center, Qingdao 266003, China; 2 Gout laboratory, The Affiliated Hospital of Qingdao University, Qingdao 266003, China; 3 State Key Laboratory of Stem Cell and Reproductive Biology, Institute of Zoology, Chinese Academy of Sciences, Beijing 100101, China

## Abstract

Chromosome 4q25 has been identified as a genomic region associated with gout. However, the associations of gout with the genes in this region have not yet been confirmed. Here, we performed two-stage analysis to determine whether variations in candidate genes in the 4q25 region are associated with gout in a male Chinese Han population. We first evaluated 96 tag single nucleotide polymorphisms (SNPs) in eight inflammatory/immune pathway- or glucose/lipid metabolism-related genes in the 4q25 region in 480 male gout patients and 480 controls. The SNP rs12504538, located in the elongation of very-long-chain-fatty-acid-like family member 6 gene (*Elovl6*), was found to be associated with gout susceptibility (*P*_adjusted_ = 0.00595). In the second stage of analysis, we performed fine mapping analysis of 93 tag SNPs in *Elovl6* and in the epidermal growth factor gene (*EGF*) and its flanking regions in 1017 male patients gout and 1897 healthy male controls. We observed a significant association between the T allele of *EGF* rs2298999 and gout (odds ratio = 0.77, 95% confidence interval = 0.67–0.88, *P*_adjusted_ = 6.42 × 10^−3^). These results provide the first evidence for an association between the *EGF* rs2298999 C/T polymorphism and gout. Our findings should be validated in additional populations.

Gout is a common form of arthritis caused by the deposition of monosodium urate crystals in joints, and it affects 1–2% of adults in developed countries^1^. The prevalence and incidence of gout have risen rapidly in recent decades in China because of diet and lifestyle changes^2^. Chronic gout has considerable social and economic burdens due to the associated pain and disability, as well as the extensive comorbidities^3^. Because men have higher levels of serum uric acid, they are more susceptible to gout than women^4^.

Although the pathogenesis of gout remains unclear, genetic susceptibility is thought to be an important factor. Indeed, the biological processes underlying regulation of this disorder are assumed to involve complex interplay among genetic, environmental and lifestyle factors. Therefore, identification of a major gout susceptibility gene would be an important step toward successful molecular diagnostics and would potentially facilitate the development of targeted therapies for patients with gout. In recent genome-wide association studies (GWAS), several hyperuricemia and gout susceptibility genes have been identified^5^,^6^,^7^,^8^,^9^,^10^,^11^, including the major urate loci solute carrier family 2 (facilitated glucose transporter), member 9 (*SLC2A9*) and ATP-binding cassette, sub-family G (*ABCG2*)^9^,^12^. As expected, polymorphisms at most urate loci have also been shown to be risk factors for gout^9^,^10^,^11^, and only four loci (*INHBB, HNF4G, UBE2Q2* and *BCAS3*) have not yet been formally associated with gout at a nominal level of significance^12^.

More recently, we have conducted a multistage GWAS in the Han Chinese population and have identified three new risk loci, 17q23.2 (*BCAS3*), 9p24.2 (*RFX3*), and 11p15.5 (*KCNQ1*), which are most likely related to progression from hyperuricemia to inflammatory gout^13^. Despite this progress, it is likely that many other unidentified genes contribute to the formation of monosodium urate crystals and clinical presentation of acute gout arthritis and chronic tophaceous disease. Genome-wide linkage studies of large families with follow-up fine mapping are considered particularly effective for identifying variants with large effects. For instance, genome-wide linkage analysis was performed on 21 multiplex pedigrees with gout from Taiwan, and chromosome 4q25 (logarithm of odds = 4.29) was found to be strongly associated with gout^14^. Additionally, our family-based linkage analysis indicated the maximum LOD score reached 1.50 at marker D4S1572 (at recombination fraction θ = 0.00), which is adjacent to the 4q25 region^15^. Therefore, we speculate that a certain gene in 4q25 region may play a role in gout susceptibility.

Within the chromosome 4q25 region, several genes have been shown to participate in regulating inflammatory/immune pathways or glucose/lipid metabolism^16^,^17^,^18^,^19^,^20^,^21^,^22^,^23^,^24^. Accordingly, several studies have demonstrated that the inflammatory/immune response and glucose/lipid metabolism play important roles in the pathogenesis of gout and that variants in the associated genes are related to this disease. *SLC2A9* encodes a member of the glucose transporter family, and variations in this gene have been previously shown to be the most significant genetic determinants of the serum urate level, accounting for 3.4–8.8% of the variance in women and for 0.5–2.0% of the variance in men^25^,^26^. The myosin light chain 2 gene (*MYL2*) encodes a regulatory light chain associated with high-density lipoprotein cholesterol metabolism, and Hirotaka *et al*. reported that the single-nucleotide polymorphism (SNP) rs2188380 of *MYL2*–*CUX2* was associated with gout in a Japanese male population^27^. Additionally, our preliminary data have shown that SNPs of inflammatory cytokine genes, such as interleukin (*IL)-8* and *IL-12B*, are associated with an increased risk of gout^28^.

In this study, we focused mainly on candidate genes in the 4q25 region that are involved in inflammatory/immune pathways or glucose/lipid metabolism. We performed a two-stage, case–control association study to determine the genetic contributions of these genes to the risk of gout in the Chinese Han population.

## Results

### Candidate SNP selection and genotyping

We selected eight candidate genes (Supplementary Note, Supplementary Table S1 and Supplementary Fig. S1), three of which encode proteins that play roles in inflammatory/immune pathways: epidermal growth factor (*EGF*); lymphoid enhancer-binding factor 1 (*LEF1*); and oligosaccharyltransferase complex subunit (*OSTC*). The other five are involved in the regulation of glucose and lipid metabolism: cytochrome P450, family 2, subfamily U, polypeptide 1 (*CYP2U1*); elongation of very-long-chain-fatty-acid-like family member 6 (*ELOVL6*); phospholipase A2, group 12 (*PLA2G12A*); sphingomyelin synthase 2 (*SGMS2*); and hydroxyacyl-CoA dehydrogenase (*HADH*).

In the first stage of analysis, 96 tag SNPs in the eight candidate genes in the 4q25 region were genotyped in 480 male gout patients and 480 matched controls (Supplementary Table S1). SNPs with call rates below 90% and *P*_HWE_ < 0.001 were excluded from subsequent analysis. A total of 91 SNPs and 960 subjects passed this initial quality control stage, and the genotype and allele frequencies are shown in Supplementary Table S2, along with the association analysis results. After correction for multiple testing, the SNP rs12504538 in *Elovl6* was found to be significantly associated with gout (*P*_adjusted_ = 0.00595).

### Association of *EGF* with gout in Han Chinese

In the second stage, we attempted to select genes upstream and downstream of *Elovl6* as the target region for fine mapping analysis. However, because the gene downstream of *Elovl6* is a pseudogene, the upstream genes *EGF* and *Elovl6* were chosen as candidate genes for further analysis. MassArray system was used to genotype 93 tag SNPs in *EGF* and *Elovl6* in an additional independent population of 1017 male gout patients and 1897 matched controls. The 91 SNPs that met the criteria (call rate > 90%, MAF > 0.05 and *P*_HWE_ > 0.001) were included in subsequent analysis (Supplementary Table S3). Power analysis revealed a power of over 80% for detecting an association at a relative risk of 1.4–1.6 (for heterozygotes and homozygotes) using an additive model.

We identified a significant association between the T allele (minor) of *EGF* rs2298999 and gout in our Chinese Han population (odds ratio (OR) = 0.77, 95% confidence interval (CI) = 0.67–0.88, *P*_adjusted_ = 6.42 × 10^−3^). Statistical analysis using the Cochran–Armitage trend test showed similar patterns (Table 1). No significant differences in allele frequencies between the gout patients and controls were detected for any of the other SNPs investigated (Supplementary Table S3). To improve the validity and reliability of this test, an additional 419 gout samples were collected from Shandong. Analysis performed after combining these data with the second stage data revealed a significant association between rs2298999 and gout (*P*_adjusted_ = 1.80 × 10^−3^, Supplementary Table S4).

**Figure 1:**
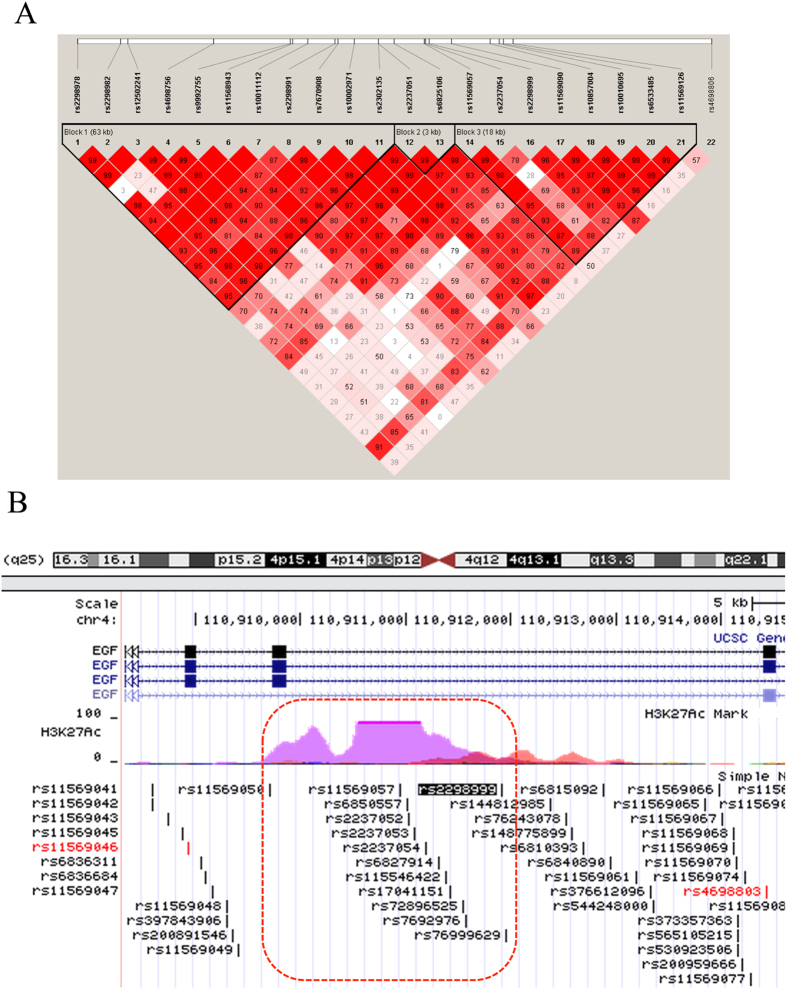
(**a**) Linkage disequilibrium (LD) analysis of single nucleotide polymorphisms (SNPs) in regions upstream and downstream of rs2298999. The rs numbers correspond to the SNP IDs and the numbers in squares refer to D’ values. The measure of LD (D’) among all possible pairs of SNPs is shown graphically according to the shade of red, where white represents very low D’ and dark red represents very high D’. (**b**) ENCODE data displayed in the UCSC database indicate that rs2298999 and 11 other SNPs are located near a region with a H3K27ac signal.

Next, linkage disequilibrium (LD) analysis was used to investigate whether any *EGF* haplotype was correlated with gout. As shown in Fig. 1a, rs2298999 was located near seven other SNPs (rs11569057, rs2237054, rs11569090, rs10857004, rs10010695, rs6533485 and rs11569126) within a block. Table 2 shows that these eight SNPs form six common haplotypes (H1–H6), with frequencies of 0.324, 0.157, 0.133, 0.125, 0.109, and 0.044, respectively, among the gout patients, and 0.281, 0.157, 0.157, 0.12, 0.114, and 0.057, respectively, among the controls. The results of the haplotype association analysis were consistent with the single SNP analysis. The risk haplotype H1 carried the risk C allele of rs2298999, and was found to be the most frequent haplotype in our analysis (32.4% in gouts, 28.1% in controls). While, the three other haplotypes (H2, H4 and H5), also carrying the C risk allele, were observed with similar frequencies between gout and control groups. Taken together, the H1 was the haplotype that showed the most significant association with gout (*P*_adjusted_ = 0.027).

Furthermore, ENCODE data from the UCSC database indicated that SNP rs2298999 is located in a region with 11 other SNPs with histone H3 acetylation at lysine 27 (H3K27ac) (Fig. 1b), which is a marker of active enhancers. This suggests that rs2298999 is located within a functional region. No significant eQTLs were found for SNP rs2298999 in all eQTL tissues in GTex database^29^, and we considered that further work is needed to determine the effect of rs2298999 on the expression of *EGF* gene.

We also investigated the potential associations between specific genotypes and different clinical indices of gout. An increase in the mean (±SD) disease duration was found to be correlated with increased mutation severity; the duration was 4.72 (±4.79) years in the T/T group compared with 5.12 (±5.42) years and 6.38 (±6.91) years in the C/T and C/C groups, respectively. This increased disease duration was found to be significant using the Kruskal–Wallis test (*P* = 0.023). Detailed findings of inferential analysis are shown in Supplementary Table S5.

## Discussion

In this study, we tested the hypothesis that candidate genes in the chromosome 4q25 region are associated with gout. Our results have shown that the *EGF* SNP rs2298999 is significantly associated with gout in the Han Chinese population and that one common *EGF* haplotype is associated with this disease.

*EGF* encodes a key growth factor that is a ligand of EGF receptor. It spans approximately 99 kb, has 24 exons and 23 introns, and encodes a 53–amino acid, single-chain polypeptide. Recently, EGF has been increasingly considered to be a pro-inflammatory mediator of many diseases, and genetic variations in this gene have also been linked to disease susceptibility^30^. Wang *et al*.^19^ have found that the *EGF* rs11568835 G/A polymorphism is associated with an increased risk of rheumatoid arthritis in the Chinese population. Moreover, a previous study has reported that gout and rheumatoid arthritis appear to have similar autoimmune mechanisms and that they share a common genetic background. Thus, our results may provide new support for the function of EGF as a pro-inflammatory mediator in gout

Our results indicated that the carriers of the *EGF* rs2298999 TT genotype in the patient group were less susceptible to gout than the carriers of the CC genotype. This SNP is located in intron 18 of *EGF*, and preliminary bioinformatics analysis has revealed that it is located with 11 other SNPs in a region with H3K27ac. This modification is generally considered to reflect active gene transcription. Emerging evidence suggests a key role of epigenetics in human inflammatory diseases; however, further study is needed to determine whether rs2298999 affects *EGF* transcription and whether these effects play a role in the development of gout.

Many genes surrounding the 4q25 region have been associated with gout, including several with reported associations in specific populations. For instance, three SNPs (rs11726117, rs231247 and rs231253) in *ALPK1* gene located in chromosome 4q21–31 were reported to be significantly associated with gout in Taiwanese populations^31^. However, these associations were not observed in a Japanese population (*P* = 0.44)^32^. The three SNPs in *ALPK1* are in high linkage disequilibrium (r^2^ = 0.964 for rs11726117 and rs231247, r^2^ = 0.964 for rs11726117 and rs231253, r^2^ = 1 for rs231253 and rs231247, from 1000 genome pilot 1 data (CHB))^33^, and among the three SNPs of *ALPK1*, rs11726117 (M861T) is the only missense SNP. Therefore, rs11726117 was genotyped in our study population of 1017 gout patients and 1897 controls, and was evaluated for a possible association with gout. Similarly, no significant association was observed between rs11726117 and gout in our population (*P* = 0.587, OR = 1.03, 95% CI = 0.92–1.15). The gene encoding cGMP-dependent protein kinase type II (*cGKII*), located at 4q13.1-q21.1, has also been shown to be associated with gout in a population of Fukien–Taiwanese heritage^34^, but no replication studies have detected an association between this gene and gout among individuals of other ancestries^35^. In addition, the SNP rs2231142 Q141K in *ABCG2,* another gene located in this region, was first reported to be associated with the serum urate level in a GWAS conducted by Dehghan and colleagues^36^. However, renal urate transporter gene variants explain only approximately 5.3% of the total variation in the serum urate concentration in the Caucasian population^36^,^37^. In the present study, we excluded these previously investigated genes located in the 4q25 region from our analysis. We focused only on genes involved in inflammatory/immune- or glucose/lipid metabolism-related pathways with the aim of identifying potentially novel genes associated with gout susceptibility in the Chinese Han population.

One key strength of our study is the relatively large sample size, which enhances detection of associations between SNPs and disease. Assuming an additive model, our study had over 80% power for detecting an association, with a genotype relative risk of 1.4–1.6 (for heterozygotes and homozygotes) at 0.05 significance. However, our study also had the following limitations: (i) it was conducted only on a Chinese Han population and should therefore be repeated for other populations to establish the link between *EGF* and gout; and (ii) we did not investigate how the genetic variation rs2298999 causes structural and functional changes to the EGF protein. The second limitation is outside the scope of this study and should be addressed in future work. To avoid the possibility that the effects of rs2298999 was overestimated due to the “winner’s curse” effect, we suggested that a larger population-based study was still needed to precisely estimate the effects and gene actions on gout.

In summary, to the best of our knowledge, this study is the first to provide evidence of an association between *EGF* and gout in a Chinese Han population. Considering the biological function of EGF, we suggest that *EGF* is a potentially novel candidate gene for gout. These findings increase the current understanding of the genetic architecture of gout; however, further study is needed to determine whether *EGF* can be a novel therapeutic agent for gout.

## Methods

### Study cohort

We recruited a total of 480 male gout patients and 480 male gout-free controls for the first stage of our analysis and an additional 1017 male gout patients and 1897 healthy (non-gout) male controls for the fine mapping stage (Supplementary Table S6). To improve the validity and reliability of this test, an additional 419 gout samples were included. All patients and controls were unrelated individuals of self-reported Han Chinese ethnicity. The diagnosis of gout was based on the preliminary Gout Classification Criteria published by the American College of Rheumatology in 1977 for use in either the clinical setting or in population-based epidemiologic studies^38^. This study was approved by the Ethics Committee of the Affiliated Hospital, Qingdao University and conducted in accordance with the ethical guidelines of the 1975 Declaration of Helsinki. All subjects gave written informed consent.

### Genotyping

A 2-mL peripheral blood sample was collected from each study participant into an EDTA tube. Genomic DNA was extracted using a whole-blood DNA isolation kit (Bioteke, Beijing, China). SNPs were selected from candidate genes in the 4q25 genomic region (the cytoBand table in the UCSC Table Browser defines 4q25 as the genomic sequence in chr4:107700000-114100000, hg19).

Samples were genotyped in the first stage of analysis using Illumina GoldenGate assay and Illumina BeadStudio software (version 3.1.0.0) according to the manufacturer’s instructions (Illumina, Inc., San Diego, CA). The original raw genotype dataset contained genotyping information for 960 samples and 96 SNPs. Following automatic clustering, the SNPs were ranked according to their GenTrain scores (from 0 to 1). Those with GenTrain scores of 0.5 were manually checked and adjusted according to the Illumina guidelines. Samples and SNPs with call rates below 90% were excluded.

In the second stage of analysis, 93 tag SNPs in *EGF* and *Elovl6* were selected for genotyping using a MassArray system (Sequenom, San Diego, CA). All procedures were performed according to the manufacturer’s instructions. Approximately 15 ng of genomic DNA was amplified by multiplex polymerase chain reaction, and then the products were used in locus-specific single-base extension reactions. The resulting products were desalted and transferred to a 384-element SpectroCHIP array. Allele detection was accomplished using matrix-assisted laser desorption/ionization time-of-flight mass spectrometry, and mass spectrograms were analyzed using MassArray TYPER software (Sequenom). For quality control, analysis was repeated for 10% of the samples, which were randomly selected.

### Statistical analysis

All SNPs were tested for Hardy–Weinberg equilibrium (HWE) in the control populations for both stages. Any SNPs that deviated from HWE (*P* < 0.001) were excluded from subsequent analyses. The genotype and allele frequencies for the cases and controls were assessed using the χ^2^ test based on 2 × 3 and 2 × 2 contingency tables. Additionally, the Cochran–Armitage trend test was used for categorical data analysis. Because the incidence of gout is related to both age and body mass index (BMI), we used age and BMI as covariates.

The associations of candidate gene SNPs with gout were evaluated using the PLINK toolset^39^. In inferential analysis, ORs, 95% CIs, and the corresponding *P* values were used to detect associations between the different genotypes and the presence of disease. All significant associations were corrected for multiple testing using the max(T) permutation procedure for both stages (100,000 permutations). Haplotype reconstruction and haplotype-based association tests were performed using the Haploview program. Individual haplotypes and their estimated population frequencies were inferred using the default parameters, and *P* values adjusted for multiple testing using the permutation procedure (100,000 permutations) were also assessed. One-way analysis of variance was performed to assess the impacts of the genotypes on the various clinical indices at presentation (alternatively, the Kruskal–Wallis test was used when indicated). The threshold *P* value was set to 0.05, and a *P* < 0.05 was considered significant.

The LD plot was generated using the Haploview program^40^, and the blocks were defined using a custom settings: CI minima for strong LD (lower, 0.7; upper, 0.95), upper CI maximum for strong recombination (0.9), and fraction of strong LD in informative comparisons (≥0.7). Markers with a frequency <0.05 were excluded.

### Power analysis

We estimated the statistical power of this study using the Genetic Power Calculator program^41^ (http://pngu.mgh.harvard.edu/~purcell/gpc/qtlassoc.html) and the following genetic model: a 22% risk allele frequency (similar to the minor allele frequency of rs2298999 in our study); and a 1.14% prevalence of gout in the Chinese population.

## Additional Information

**How to cite this article**: Han, L. *et al*. Epidermal growth factor gene is a newly identified candidate gene for gout. *Sci. Rep.* **6**, 31082; doi: 10.1038/srep31082 (2016).

## Supplementary Material

Supplementary Information

## Figures and Tables

**Table 1:** rs2298999 in *EGF* gene associated with gout in the second stage.

	Controls	Gouts	Pcrude	Padjusted
Genotype				
CC	1077(57.01)	647(64.38)		
CT	709(37.54)	325(32.24)		
TT	103(5.45)	36(3.38)	3.9*10^−4^	0.025
Allele				
C	2863(75.78)	1619(80.30)		
T	915(24.22)	397(19.70)	8.80*10^−5^	6.42*10^−3^
Trend				
C	2863(75.78)	1619(80.30)		
T	915(24.22)	397(19.70)	7.53*10^−5^	5.18*10^−3^

Notes: Trend: Cochran-Armitage trend test.

**Table 2:** Haplotype-based association analysis of tagging SNPs in *EGF* gene.

	Haplotypes	Controls (%)	Gouts (%)	Chi-Square	*P*_crude_	*P*_adjusted_
H1	ATCGTACG	28.1	32.4	11.851	6.0*10^−4^	0.027
H2	AACGTACA	15.7	15.7	0	0.997	1
H3	ATTGCGGG	15.7	13.3	5.932	0.015	0.551
H4	GACGTACG	12	12.5	0.376	0.54	1
H5	ATCGCGCG	11.4	10.9	0.262	0.609	1
H6	ATTACGGG	5.7	4.4	4.486	0.034	0.8847

Notes: The haploypes listed here were composed of rs11569057, rs2237054, rs2298999, rs11569090, rs10857004 rs10010695, rs6533485 and rs11569126.
