# Supplementary material for: Epidermal growth factor gene is a newly identified candidate gene for gout
Source: Sci Rep. 2016 Aug 10;6:31082. doi: 10.1038/srep31082 (PMC4978989; doi:10.1038/srep31082)
Supplement: Supplementary Information [file srep31082-s1.pdf]

Epidermal growth factor gene is a newly identified candidate gene for gout

Lin Han[1,2,] ,Chunwei Cao[3,],Zhaotong Jia[1,2,],Shiguo Liu[1,2,],Zhen Liu[1,2,], Ruosai Xin[1,2,], Can Wang [1,2,], Xinde Li[1,2,],Wei Ren[1,2,],Xuefeng Wang[1,2,], Changgui Li[1,2*]

[1] Shandong Gout Clinical Medical Center, Qingdao 266003, China. [2] Gout laboratory, The Affiliated Hospital of Qingdao University, Qingdao 266003, China. [3] State Key Laboratory of Stem Cell and Reproductive Biology, Institute of Zoology, Chinese Academy of Sciences, Beijing 100101, China.

Lin Han and Chunwei Cao contributed equally to this work.

Correspondence and requests for materials should be addressed to C.L. (email:lichanggui@medmail.com.cn)

Supplementary Note: Strategy for candidate gene selection

Gout, the most common form of inflammatory arthritis, is caused by hyperuricemia and has substantial morbidity associated with excruciating pain. It is widely accepted that an elevated uric acid level and monosodium urate crystal-induced inflammation are critical risk factors for gout pathology[1-3].

A high circulating serum urate concentration has been shown to be associated with dysmetabolism, including abdominal obesity, impaired fasting glucose, hypertriglyceridemia, a low high-density lipoprotein cholesterol level, and elevated blood pressure[4-6]. Although

considerable evidence indicates that the serum uric acid level is strongly associated with fatty acid synthase, the underlying mechanism remains unknown. However, several pieces of evidence support various hypotheses: first, uric acid-induced endoplasmic reticulum stress may result in an increase in fatty acid synthase via activation of the gene encoding sterol-regulatory element binding protein 1c (*SREBP-1c*)[7]. SREBP-1c is a well-known driver of lipogenesis that activates its lipid synthetic downstream targets, fatty acid synthase and acetyl-CoA carboxylase[8]. In addition to SREBP-1c, uric acid may also directly stimulate hepatic fat accumulation, citrate accumulation, and fat synthesis[9].

The role of genetic polymorphisms in the link between hyperuricemia and nonalcoholic fatty liver disease (NAFLD) has been investigated in recent years. An *ABCG2* polymorphism, rs2231142, has been reported to be a significant predictor of both the serum uric acid level and NAFLD[10]. Two other major ABC efflux transport proteins, ABCC1-6 and ABCB1, have also been shown to exhibit increasing trends with NAFLD progression[11]. Therefore, in the present study, we selected SNPs in genes located in the 4q25 genomic region, including *CYP2U1*[12,13], *ELOVL6*[14], *PLA2G12A*[15,16], and *SGMS2*[17,18], which are involved in the hydroxylation, catalysis, synthesis, uptake, and production of fatty acids, respectively.

It has also been hypothesized that insulin resistance may affect the serum uric acid level. Elevated serum uric acid levels have been detected in individuals with hyperinsulinemia[19], while physiological hyperinsulinemia acutely reduces the urinary uric acid level and sodium excretion in a coupled manner[20]. In our study, we chose *HADH* as a candidate gene because its reported mutations are the most common causes of congenital hyperinsulinism[21,22].

The immune inflammatory response has also been reported to be closely associated

with the pathology of gout. Indeed, gout inflammation is thought to be triggered by immune interactions between monosodium urate crystals and the local tissue environment. Specifically, deposition of monosodium urate crystals can promote inflammation by reacting with immune cells, such as macrophages, monocytes, and neutrophils. Further, recruitment of inflammatory leukocytes to local tissues (e.g., joints) can lead to the subsequent release of inflammatory mediators and cause the recognized inflammatory manifestations of acute gout[23,24]. In this study, we investigated the correlations between gout and select candidate genes (*EGF*[25,26], *LEF1*[27], and *OSTC*[28]) located in the 4q25 region that have been previously reported to play roles in inflammatory or immune responses. For instance, one study has demonstrated that EGF synergistically stimulates IL-8 production in the presence of IL-1β, while synergistic interactions between EGF and IL-1β in promoting IL-8 production may play an essential role in the pathogenesis of inflammatory disease[29]. Additionally, LEF-1 and T-cell factor 1 have been demonstrated to be important for early thymocyte maturation beyond the $CD4^-CD8^-$ stage; they have also been reported to act upstream of Th-POK to promote the CD4 (+) T cell fate[30, 31]. Finally, OSTC, a type of glycosyltransferase, appears to be involved in glycosylation, which has been reported to contribute to the inflammatory response[28].

Table S1. Candidate genes, putative roles for selection and reference list.

Genes	Position (hg19)	Functional role	References
CYP2U1	chr4:108852717-108874613	fatty acid metabolism	[12, 13]
ELOVL6	chr4:110970229-111119820	fatty acid metabolism	[14]
PLA2G12A	chr4:110631145-110651242	fatty acid metabolism	[15, 16]
SGMS2	chr4:108745721-108836204	fatty acid metabolism	[17, 18]
HADH	chr4:108910870-108956331	glucose metabolism (hyperinsulinemia)	[21, 22]
EGF	chr4:110834040-110934118	inflammation response	[25, 26, 29]
LEF1	chr4:108968701-109090112	inflammation response	[27, 30, 31]
OSTC	chr4:109571741-109588978	inflammation response	[28]

Table S2. SNPs successfully genotyped in the first stage (polymorphisms in chromosome 4q25 candidate genes)

Gene symbol	SNP	BP	Major Allele	Minor Allele	MAF-case	MAF-con	X^2	OR	95% CI	P_{crude}	$P_{adjusted}$	P_{trend}
SGMS2	rs10021742	108746990	C	T	0.2197	0.2197	1.77E-05	1	0.8051-1.243	0.9966	1	1
SGMS2	rs868071	108753639	G	A	0.2489	0.2469	0.01108	1.011	0.8213-1.245	0.9162	1	1
SGMS2	rs11940819	108788680	A	G	0.2574	0.2406	0.7184	1.094	0.8887-1.347	0.3967	1	1
SGMS2	rs17038142	108792964	A	G	0.2685	0.2537	0.5415	1.08	0.8799-1.325	0.4618	1	1
SGMS2	rs6854068	108801540	G	C	0.3407	0.3044	2.874	1.181	0.9743-1.432	0.08999	0.9894	0.9872
SGMS2	rs12648528	108802762	T	G	0.351	0.3302	0.9118	1.097	0.9072-1.326	0.3396	1	1
SGMS2	rs17038197	108815600	G	A	0.193	0.1904	0.02176	1.017	0.8097- 1.278	0.8827	1	1
SGMS2	rs2131462	108819891	G	C	0.1245	0.08996	5.927	1.438	1.072-1.929	0.01491	0.5493	0.6166
CYP2U1	rs4395588	108860978	G	A	0.3112	0.273	3.354	1.203	0.987-1.466	0.06703	0.9661	0.9704
HADH	rs2267842	108927740	C	T	0.3112	0.3232	0.3186	0.9459	0.7798-1.147	0.5725	1	1
LEF1	rs13109482	108983032	T	G	0.08966	0.06695	3.405	1.373	0.9794-1.924	0.06498	0.9625	0.9653
LEF1	rs922166	109060567	C	T	0.3903	0.363	1.514	1.123	0.9333-1.352	0.06498	1	1
LEF1	rs12503104	109062510	C	T	0.2711	0.2615	0.2241	1.05	0.8571-1.287	0.6359	1	1
OSTC	rs2851374	109588238	G	C	0.3422	0.3431	0.001844	0.9959	0.8239-1.204	0.9657	1	1
PLA2G12A	rs2285714	110638810	C	T	0.2215	0.25	2.143	0.8537	0.6906-1.055	0.1433	0.9993	0.9995
PLA2G12A	rs6533451	110647195	G	A	0.461	0.4059	5.888	1.252	1.044-1.501	0.01525	0.5598	0.6075
PLA2G12A	rs10004377	110648124	T	G	0.4209	0.3839	2.709	1.166	0.971-1.401	0.09977	0.9933	0.9959
PLA2G12A	rs6854876	110653440	C	G	0.3418	0.3494	0.1216	0.967	0.8005-1.168	0.7273	1	1
EGF	rs3756267	110831276	T	C	0.1498	0.1355	0.7926	1.124	0.8689-1.454	0.3733	1	1
EGF	rs3756265	110831635	G	A	0.1617	0.1548	0.171	1.053	0.8234-1.348	0.6792	1	1
EGF	rs2250724	110837287	C	T	0.2352	0.2333	0.01028	1.011	0.8178-1.25	0.9192	1	1
EGF	rs2298977	110837573	C	A	0.07384	0.05962	1.545	1.257	0.8757-1.806	0.2138	1	1
EGF	rs2298978	110837667	G	A	0.4167	0.3912	1.281	1.112	0.9255-1.335	0.2577	1	1

EGF	rs2298982	110846796	A	G	0.3481	0.3337	0.4405	1.066	0.8822-1.289	0.5069	1	1
EGF	rs3822288	110854544	G	A	0.2078	0.1904	0.907	1.116	0.8907-1.397	0.3409	1	1
EGF	rs11568875	110860818	G	A	0.08245	0.05241	6.823	1.625	1.126-2.345	0.009	0.388	0.3767
EGF	rs7655579	110865708	A	G	0.4694	0.4791	0.1785	0.962	0.8036-1.152	0.6726	1	1
EGF	rs11568941	110882980	G	T	0.3217	0.3201	0.00592	1.008	0.8312-1.221	0.9387	1	1
EGF	rs1860129	110886343	C	G	0.288	0.3033	0.5401	0.9288	0.7628-1.131	0.4624	1	1
EGF	rs2255355	110891543	C	A	0.3586	0.3536	0.05385	1.022	0.8476-1.233	0.8165	1	1
EGF	rs2237050	110900818	A	G	0.1424	0.1313	0.4957	1.099	0.8455-1.427	0.4814	1	1
EGF	rs2298995	110905976	A	C	0.2963	0.3166	0.9037	0.909	0.7467-1.107	0.3418	1	1
EGF	rs2237052	110911025	G	A	0.296	0.3044	0.1601	0.9608	0.7897-1.169	0.6891	1	1
EGF	rs11569090	110916636	G	A	0.09283	0.08787	0.1426	1.062	0.7764-1.453	0.7058	1	1
EGF	rs4698757	110922194	T	A	0.1646	0.1572	0.1889	1.056	0.9041-1.297	0.6638	1	1
EGF	rs3733627	110925469	G	A	0.4747	0.4549	0.7461	1.083	0.9041-1.297	0.3877	1	1
EGF	rs6533485	110927563	C	G	0.2584	0.2542	0.04521	1.023	0.8324-1.256	0.8316	1	1
EGF	rs971696	110927865	A	T	0.3755	0.3755	3.95E-08	1	0.8307-1.204	0.9998	1	1
EGF	rs2074391	110929729	G	A	0.1804	0.1559	2.047	1.192	0.9369-1.516	0.1525	0.9995	0.9996
EGF	rs3733625	110933004	A	G	0.1762	0.173	0.03389	1.022	0.8069-1.296	0.854	1	1
ELOVL6	rs4141123	110973665	A	G	0.37	0.3556	0.4224	1.064	0.8825-1.283	0.5157	1	1
ELOVL6	rs7662161	110975433	T	C	0.212	0.2176	0.08686	0.9676	0.7775-1.204	0.7682	1	1
ELOVL6	rs7690709	110976538	C	A	0.3407	0.3692	1.692	0.8828	0.7316-1.065	0.1933	1	0.9999
ELOVL6	rs17041301	110976758	C	T	0.1508	0.1684	1.095	0.8772	0.6861-1.121	0.2954	1	1
ELOVL6	rs17041304	110977348	T	G	0.1582	0.1485	0.3444	1.078	0.8397-1.383	0.5573	1	1
ELOVL6	rs12505992	110981940	C	A	0.4293	0.4226	0.08819	1.028	0.8571-1.233	0.7665	1	1
ELOVL6	rs7683007	110982130	G	C	0.3291	0.3358	0.09514	0.9704	0.8019-1.174	0.7577	1	1
ELOVL6	rs17041323	110984555	G	A	0.3629	0.3964	2.278	0.8671	0.7204-1.044	0.1312	0.9988	0.9983
ELOVL6	rs17041324	110984714	A	T	0.2294	0.2437	0.5411	0.9237	0.7475-1.141	0.462	1	1

ELOVL6	rs17041326	110986845	C	T	0.1593	0.1454	0.7108	1.114	0.8671-1.43	0.3992	1	1
ELOVL6	rs16997129	110989719	T	C	0.2859	0.2772	0.1768	1.044	0.8548-1.275	0.6741	1	1
ELOVL6	rs17041343	110994069	G	A	0.2975	0.3316	2.57	0.8535	0.7032-1.036	0.1089	0.9961	0.9953
ELOVL6	rs4698807	110994223	C	G	0.3376	0.3033	2.558	1.17	0.9651-1.419	0.1098	0.9963	0.9962
ELOVL6	rs10033691	110995710	T	C	0.1814	0.1831	0.00838	0.9892	0.7838-1.248	0.9271	1	1
ELOVL6	rs11731118	111004093	T	A	0.2215	0.2316	0.274	0.9442	0.7615-1.171	0.6006	1	1
ELOVL6	rs11943595	111006785	A	G	0.4778	0.5021	1.12	0.9075	0.7582-1.086	0.29	1	1
ELOVL6	rs11731407	111011715	G	A	0.1698	0.1538	0.906	1.126	0.8819-1.437	0.3412	1	1
ELOVL6	rs6533491	111030787	C	T	0.1603	0.1481	0.5446	1.098	0.8561-1.409	0.4605	1	1
ELOVL6	rs1005595	111032048	C	T	0.4809	0.511	1.779	0.8847	0.7389-1.059	0.1823	0.9999	0.9999
ELOVL6	rs1005594	111032489	C	T	0.154	0.1559	0.01243	0.986	0.7692-1.264	0.9112	1	1
ELOVL6	rs1001857	111032602	A	G	0.3618	0.4046	3.685	0.8343	0.6933-1.004	0.05492	0.9385	0.9385
ELOVL6	rs11937052	111033020	A	G	0.1867	0.2029	0.7982	0.9017	0.7186-1.132	0.3716	1	1
ELOVL6	rs12512099	111033406	C	T	0.1319	0.159	2.82	0.8034	0.622-1.038	0.09309	0.9908	0.9918
ELOVL6	rs1315962	111033550	G	A	0.4093	0.4655	6.109	0.7956	0.6636-0.9539	0.01345	0.5136	0.4866
ELOVL6	rs1125819	111036402	G	A	0.4504	0.4132	2.691	1.164	0.9708-1.396	0.1009	0.9938	0.9881
ELOVL6	rs1026883	111036781	A	G	0.1649	0.1447	1.477	0.9636	0.9094-1.499	0.2242	1	1
ELOVL6	rs12649452	111037159	C	T	0.2416	0.2484	0.1212	0.9636	0.7818-1.188	0.7277	1	1
ELOVL6	rs11723007	111041251	C	G	0.4568	0.4498	0.09305	1.028	0.8587-1.232	0.7603	1	1
ELOVL6	rs12504798	111042340	T	C	0.2313	0.2416	0.2775	0.9446	0.7641-1.168	0.5983	1	1
ELOVL6	rs2170553	111043210	A	C	0.2468	0.2521	0.07025	0.9723	0.79-1.197	0.791	1	1
ELOVL6	rs10010167	111051486	C	T	0.4451	0.4331	0.2826	1.05	0.8764-1.259	0.595	1	1
ELOVL6	rs17539673	111052322	T	A	0.1055	0.09728	0.3518	1.094	0.8124-1.474	0.5531	1	1
ELOVL6	rs7689036	111053652	A	G	0.4209	0.3996	0.893	1.092	0.9097-1.311	0.3447	1	1
ELOVL6	rs10516560	111054395	G	C	0.09342	0.09329	9.01E-05	1.001	0.735-1.365	0.9924	1	1
ELOVL6	rs6533495	111055435	G	A	0.4451	0.4393	0.0653	1.024	0.8544-1.227	0.7983	1	1

ELOVL6	rs11936079	111055558	C	T	0.3165	0.3159	0.0006809	1.003	0.8264-1.216	0.9792	1	1
ELOVL6	rs12233783	111056933	C	T	0.1245	0.1098	0.9866	1.152	0.871-1.524	0.3206	1	1
ELOVL6	rs1979469	111060898	A	G	0.3597	0.3243	2.657	1.171	0.9685-1.415	0.1031	0.9944	0.9918
ELOVL6	rs6835929	111064092	A	G	0.3996	0.3855	0.3941	1.061	0.8823-1.275	0.5302	1	1
ELOVL6	rs4698812	111065534	T	C	0.3323	0.3358	0.02615	0.9844	0.8137-1.191	0.8715	1	1
ELOVL6	rs6813255	111072985	A	G	0.3354	0.3556	0.8593	0.9145	0.757-1.105	0.3539	1	1
ELOVL6	rs4146697	111078453	T	C	0.1797	0.1688	0.3952	1.079	0.8512-1.368	0.5296	1	1
ELOVL6	rs7682028	111082556	T	C	0.1878	0.1908	0.02811	0.9806	0.7795-1.233	0.8668	1	1
ELOVL6	rs4349664	111092061	G	A	0.1044	0.1161	0.6616	0.8877	0.6661-1.183	0.416	1	1
ELOVL6	rs1384331	111105442	A	C	0.3143	0.3075	0.1032	1.032	0.8502-1.253	0.7481	1	1
ELOVL6	rs12498965	111106956	T	C	0.1867	0.1977	0.3702	0.9316	0.7417-1.17	0.5429	1	1
ELOVL6	**rs12504538**	**111112448**	**T**	**C**	**0.1392**	**0.08368**	**14.85**	**1.771**	**1.321-2.376**	**0.0001165**	**0.00595**	**0.00633**
ELOVL6	rs6533498	111112788	C	A	0.327	0.3295	0.01342	0.9888	0.8166-1.197	0.9078	1	1
ELOVL6	rs11098071	111117247	G	A	0.3861	0.3828	0.02099	1.014	0.8428-1.219	0.8848	1	1
ELOVL6	rs6824447	111120713	G	A	0.3932	0.3912	0.008149	1.009	0.8389-1.212	0.9281	1	1
ELOVL6	rs6831073	111121841	G	A	0.1213	0.1172	0.07819	1.04	0.7884-1.373	0.7798	1	1

The chromosome coordinates of the SNPs were based on hg19.

Table S3. Fine mapping analysis of *EGF-Elove6* gene region

Gene symbol	SNP	BP	Major Allele	Minor Allele	MAF-case	MAF-con	X^2	OR	(95% CI)	P_{crude}	$P_{adjusted}$	P_{trend}
EGF	rs2298978	110837667	G	A	0.400	0.417	1.689	0.930	0.8324-1.038	0.1938	0.9996	0.9997
EGF	rs2298982	110846796	A	G	0.341	0.349	0.330	0.967	0.8635-1.084	0.5658	1	1
EGF	rs12502241	110848308	G	A	0.137	0.133	0.223	1.039	0.8872 -1.216	0.637	1	1
EGF	rs4698756	110866442	G	A	0.513	0.482	5.172	1.133	1.017-1.262	0.02295	0.6623	0.684
EGF	rs9992755	110882590	A	G	0.197	0.199	0.033	0.988	0.8625-1.13	0.8548	1	1
EGF	rs11568943	110883121	G	A	0.209	0.224	1.844	0.913	0.8002- 1.041	0.1744	0.9992	0.9994
EGF	rs10011112	110886306	G	A	0.060	0.067	1.335	0.877	0.7012-.096	0.248	1	1
EGF	rs2298991	110892012	G	T	0.218	0.241	3.929	0.878	0.7714-0.9986	0.04746	0.8775	0.8799
EGF	rs7670908	110892625	G	A	0.211	0.219	0.455	0.956	0.8374-1.09	0.5002	1	1
EGF	rs10002971	110896050	A	C	0.058	0.069	2.686	0.829	0.6625-1.038	0.1012	0.9845	0.9837
EGF	rs2302135	110901147	A	G	0.319	0.283	8.486	1.190	1.059-1.338	0.003578	0.1846	0.1884
EGF	rs2237051	110901198	A	G	0.284	0.312	4.719	0.877	0.779-0.9873	0.02984	0.7464	0.7643
EGF	rs6825106	110904502	A	G	0.344	0.381	8.053	0.850	0.7589-0.9508	0.004543	0.2249	0.2381
EGF	rs11569057	110910941	A	G	0.131	0.123	0.721	1.072	0.9127-1.26	0.3958	1	1
EGF	rs2237054	110911189	T	A	0.343	0.342	0.005	1.004	0.8963-.125	0.9411	1	1
EGF	**rs2298999**	**110911907**	**C**	**T**	**0.197**	**0.242**	**15.380**	**0.767**	**0.672-0.8761**	**8.80**$*10^{-5}$	**0.00642**	**0.00518**
EGF	rs11569090	110916636	G	A	0.058	0.073	4.343	0.789	0.6318-0.9865	0.03717	0.8123	0.8095
EGF	rs10857004	110924772	T	C	0.362	0.401	8.485	0.847	0.758-0.9473	0.003581	0.1846	0.1977
EGF	rs10010695	110926730	A	G	0.355	0.400	11.090	0.826	0.7373-0.9242	0.000869	0.05432	0.05979
EGF	rs6533485	110927563	C	G	0.240	0.277	9.382	0.824	0.7272-0.9326	0.002191	0.123	0.1176
EGF	rs11569126	110929653	G	A	0.170	0.175	0.214	0.967	0.8379-1.116	0.644	1	1
ELOVL6	rs6533495	111055435	G	A	0.449	0.434	1.192	1.062	0.953-1.185	0.5962	1	1
ELOVL6	rs4698806	110971489	G	A	0.423	0.416	0.281	1.030	0.9234-1.149	0.2833	1	1

ELOVL6	rs7684686	110975685	C	T	0.213	0.225	1.151	0.931	0.8166-1.061	0.7649	1	1
ELOVL6	rs17041301	110976758	C	T	0.142	0.139	0.089	1.024	0.8769- 1.196	0.8895	1	1
ELOVL6	rs12500716	110977136	G	A	0.143	0.144	0.019	0.989	0.848-1.154	0.2087	0.9998	0.9999
ELOVL6	rs10001138	110978130	A	C	0.330	0.314	1.580	1.077	0.9595-1.208	0.1812	0.9993	0.9996
ELOVL6	rs1557803	110978498	G	A	0.329	0.312	1.788	1.082	0.9638-1.215	0.1369	0.9962	0.9968
ELOVL6	rs72676981	110979557	G	A	0.145	0.160	2.213	0.892	0.7665-1.037	0.1092	0.9889	0.9902
ELOVL6	rs12505992	110981940	C	A	0.369	0.391	2.566	0.913	0.8158-1.021	0.05677	0.9157	0.9139
ELOVL6	rs7683007	110982130	G	C	0.337	0.312	3.629	1.118	0.9968-1.254	0.3469	1	1
ELOVL6	NA	110984370	C	T	0.149	0.140	0.885	1.077	0.9227-1.257	0.3499	1	1
ELOVL6	rs17041323	110984555	G	A	0.372	0.384	0.874	0.948	0.8483- 1.06	0.1208	0.9932	0.9938
ELOVL6	NA	110985406	G	A	0.146	0.162	2.407	0.888	0.7636-1.032	0.295	1	1
ELOVL6	rs17041330	110988287	T	C	0.237	0.249	1.097	0.935	0.8241-1.06	0.3601	1	1
ELOVL6	rs16997129	110989719	T	C	0.280	0.269	0.837	1.058	0.9377-1.194	0.2273	0.9999	0.9999
ELOVL6	rs17041343	110994069	G	A	0.311	0.326	1.458	0.931	0.8283-1.046	0.6548	1	1
ELOVL6	rs11737840	110994243	C	T	0.062	0.059	0.200	1.053	0.8399-1.32	0.2953	1	1
ELOVL6	rs10024683	110996171	A	C	0.308	0.295	1.095	1.065	0.9467-1.198	0.6209	1	1
ELOVL6	rs56338714	111006264	A	G	0.205	0.199	0.245	1.035	0.9044-1.183	0.5972	1	1
ELOVL6	rs34415611	111007345	G	A	0.204	0.199	0.279	1.037	0.9065-1.186	0.7756	1	1
ELOVL6	NA	111008395	C	T	0.143	0.140	0.081	1.023	0.8763-1.194	0.98	1	1
ELOVL6	rs28572278	111012820	T	C	0.356	0.357	0.001	0.999	0.8912-1.119	0.9911	1	1
ELOVL6	NA	111017077	T	C	0.135	0.357	0.000	0.999	0.8534-1.17	0.1865	0.9994	0.9996
ELOVL6	rs56145754	111017441	A	C	0.512	0.494	1.745	1.075	0.9654-1.198	0.5357	1	1
ELOVL6	rs61185590	111017544	G	A	0.149	0.143	0.384	1.049	0.9011-1.222	0.5132	1	1
ELOVL6	rs7674189	111020907	G	A	0.270	0.262	0.427	1.042	0.9219-1.177	0.941	1	1
ELOVL6	NA	111024853	C	T	0.139	0.140	0.005	0.994	0.8504-1.162	0.8146	1	1
ELOVL6	rs11731449	111025534	C	T	0.146	0.148	0.055	0.982	0.8431-1.144	0.8686	1	1

ELOVL6	rs72679215	111025720	T	C	0.175	0.177	0.027	0.988	0.8575-1.139	0.678	1	1
ELOVL6	rs10011926	111026927	T	C	0.268	0.262	0.172	1.026	0.9083-1.159	0.9338	1	1
ELOVL6	rs10027629	111028732	T	C	0.419	0.420	0.007	0.995	0.8924-1.11	0.9815	1	1
ELOVL6	rs1005595	111032048	G	A	0.493	0.493	0.001	0.999	0.8963-1.113	0.2608	1	1
ELOVL6	rs1005594	111032489	G	A	0.159	0.147	1.264	1.089	0.9384-1.265	0.2682	1	1
ELOVL6	rs1001857	111032602	A	G	0.356	0.371	1.226	0.939	0.8388-1.05	0.674	1	1
ELOVL6	rs11937052	111033020	A	G	0.163	0.167	0.177	0.969	0.8381-1.121	0.9317	1	1
ELOVL6	rs11098070	111033180	G	A	0.429	0.428	0.007	1.005	0.9009-1.121	0.8992	1	1
ELOVL6	rs12512099	111033406	C	T	0.154	0.153	0.016	1.010	0.8695- 1.172	0.6633	1	1
ELOVL6	rs1125819	111036402	C	T	0.417	0.411	0.190	1.025	0.9184-1.143	0.4573	1	1
ELOVL6	rs1026883	111036781	T	C	0.077	0.082	0.553	0.927	0.7586- 1.132	0.8776	1	1
ELOVL6	rs12649452	111037159	C	T	0.234	0.235	0.024	0.990	0.8717-1.124	0.4945	1	1
ELOVL6	rs2170553	111043210	A	C	0.241	0.249	0.467	0.957	0.8436-1.086	0.9192	1	1
ELOVL6	rs13145220	111047797	T	A	0.066	0.065	0.010	1.011	0.8136 -1.257	0.2506	1	1
ELOVL6	rs10010167	111051486	C	T	0.419	0.435	1.320	0.937	0.8394- 1.047	0.4961	1	1
ELOVL6	rs7689036	111053652	A	G	0.418	0.427	0.463	0.963	0.8632-1.074	0.796	1	1
ELOVL6	rs10516560	111054395	G	C	0.095	0.093	0.067	1.025	0.8524-1.232	0.2748	1	1
ELOVL6	rs11936079	111055558	C	T	0.319	0.317	0.018	1.008	0.8976- 1.132	0.8922	1	1
ELOVL6	rs7667291	111055836	G	A	0.343	0.364	2.555	0.912	0.8142- 1.021	0.11	0.9892	0.9875
ELOVL6	NA	111058377	T	C	0.188	0.188	0.000	1.000	0.8142-1.021	0.9961	1	1
ELOVL6	NA	111058693	T	C	0.066	0.054	3.357	1.233	0.8713-1.148	0.06694	0.9429	0.9357
ELOVL6	rs71603056	111059125	G	A	0.111	0.117	0.530	0.939	0.7916-1.113	0.4665	1	1
ELOVL6	rs28722886	111060511	C	T	0.302	0.314	0.874	0.946	0.8415- 1.063	0.3497	1	1
ELOVL6	NA	111065511	A	G	0.152	0.154	0.041	0.985	0.8477-1.144	0.8398	1	1
ELOVL6	rs4698812	111065534	T	C	0.344	0.316	4.727	1.137	1.013-1.277	0.0297	0.7453	0.7592
ELOVL6	rs1137543	111065632	A	G	0.189	0.167	4.283	1.161	1.008-1.338	0.0385	0.8225	0.8129

ELOVL6	rs60203471	111073058	G	A	0.171	0.170	0.024	1.011	0.8763-1.167	0.8776	1	1
ELOVL6	rs72900544	111074088	G	A	0.156	0.154	0.039	1.015	0.8743-1.179	0.8427	1	1
ELOVL6	NA	111076359	C	T	0.188	0.171	2.550	1.122	0.9741-1.293	0.1103	0.9894	0.9883
ELOVL6	rs34505563	111077109	A	G	0.125	0.127	0.086	0.976	0.8295-1.148	0.7696	1	1
ELOVL6	rs4146698	111078326	A	G	0.349	0.331	1.868	1.083	0.9662-1.213	0.1717	0.9991	0.9991
ELOVL6	rs2200106	111079337	G	A	0.148	0.142	0.393	1.050	0.9012-1.224	0.5305	1	1
ELOVL6	rs7681062	111082170	T	C	0.198	0.177	3.910	1.149	1.001-1.319	0.04799	0.8803	0.8813
ELOVL6	rs12498965	111106956	T	C	0.172	0.180	0.502	0.950	0.8239-1.095	0.4788	1	1
ELOVL6	rs13145689	111109020	A	G	0.314	0.319	0.150	0.977	0.8702-1.098	0.6987	1	1
ELOVL6	NA	111109702	C	T	0.083	0.091	0.933	0.909	0.7493-1.103	0.334	1	1
ELOVL6	NA	111109721	G	T	0.092	0.089	0.112	1.033	0.855-1.248	0.7375	1	1
ELOVL6	rs62327360	111111589	G	A	0.088	0.087	0.001	1.003	0.8291- 1.214	0.973	1	1
ELOVL6	rs12504538	111112448	T	C	0.096	0.098	0.059	0.978	0.8144-1.173	0.8075	1	1
ELOVL6	rs6533498	111112788	C	A	0.333	0.321	0.945	1.059	0.9437-1.188	0.331	1	1
ELOVL6	rs11098071	111117247	G	A	0.395	0.396	0.001	0.998	0.8932-1.115	0.9696	1	1
ELOVL6	rs3813828	111120148	A	G	0.070	0.078	1.226	0.889	0.7226-1.095	0.2682	1	1

NA: not in dbSNP database. Odds ratios, 95% CI and p-values are derived from the allelic test for association using X^2 test (1 df). The significances (including p-values from Cochran-Armitage trend test) were corrected for multiple testing using max(T) permutation procedure (100,000 permutations). The chromosome coordinates of the SNPs were based on hg19.

Table S4. Association analysis of rs2298999 in extended samples

Allele	Controls	Gouts	$P_{adjusted}$	OR	95% CI
C	2863(75.78)	2255 (79.00)	$1.80*10^{-3}$	0.83	0.74-0.94
T	915(24.22)	599 (21.00)			

Notes : extended samples, an additional 419 gout samples were added to second stage samples; OR, odds ratio; CI, confidence interval; The significancewere corrected for multiple testing using max(T) permutation procedure (100,000 permutations).

Table S5. Comparison of various quantitative phenotypes among the different genotypes at rs2298999 in *EGF* gene

	C/C<br>n=647	C/T<br>n=325	T/T<br>n=36	*P*-values
Demographic characteristics				
Age (yr)	52.83±13.06	52.14±13.11	51.32±12.28	0.634
Age at diagnosis (yr)	46.57±12.82	47.11±12.90	46.47±12.35	0.824
Disease duration (yr)	6.38±6.91	5.12±5.42	4.72±4.79	**0.023**
Tophi	137/560	58/283	8/28	0.349
BMI (kg/m2)	27.18±5.03	26.91±4.17	27.54±2.73	0.634
WHR	0.96±0.05	0.93±0.06	0.93±0.05	0.337
Systolic pressure (mmHg)	137.46±20.68	137.17±20.09	136.03±17.47	0.916
Diastolic pressure (mmHg)	89.11±13.72	90.03±13.04	92.27±10.26	0.3
Serum Biochemistry				
Blood Glucose (mmol/L)	6.16±1.63	6.17±1.61	5.80±1.42	0.439
Uric acid (umo1/L)	469.28±111.51	456.62±107.66	492.22±108.88	0.092
Triglycerides (mmol/L)	2.40±1.74	2.74±5.96	3.09±4.32	0.277
Total cholesterol (mmol/L)	5.23±1.14	5.27±1.19	5.41±1.31	0.663
Urea nitrogen (mmol/L)	5.99±4.56	5.58±2.35	5.56±1.97	0.324
Creatinine (umo1/L)	91.53±35.32	90.40±62.96	90.30±29.68	0.937
ALT (U/L)	35.47±25.21	32.63±24.81	28.49±10.26	0.279
AST (U/L)	27.32±16.37	26.38±12.42	24.63±9.46	0.638
Past history				
hypertension	279/630	141/316	21/33	0.092
Diabetes	48/622	37/314	2/34	0.098
Coronary atherosclerosis	83/618	44/314	3/33	0.732

Hyperlipidemia	182/502	90/262	12/27	0.561
Kidney disease	38/620	16/315	2/33	0.808
Kidney stones	135/548	61/271	7/28	0.792

Notes: BMI, Body mass index; WHR, waist to hip ratio.

Table S6. Description of the first stage and second stage samples

	N	Age (years±s.d.)	BMI	Acid Uric (umoll^{-1}±s.d.)
first stage				
Cases	480	52.84±13.12	26.87±3.46	495.98±133.08
Controls	480	60.57±9.45	24.78±2.90	322.94±56.90
second stage				
Cases	1017	52.10±12.82	27.21±3.98	472.71±109.35
Controls	1897	53.9±7.92	-	-

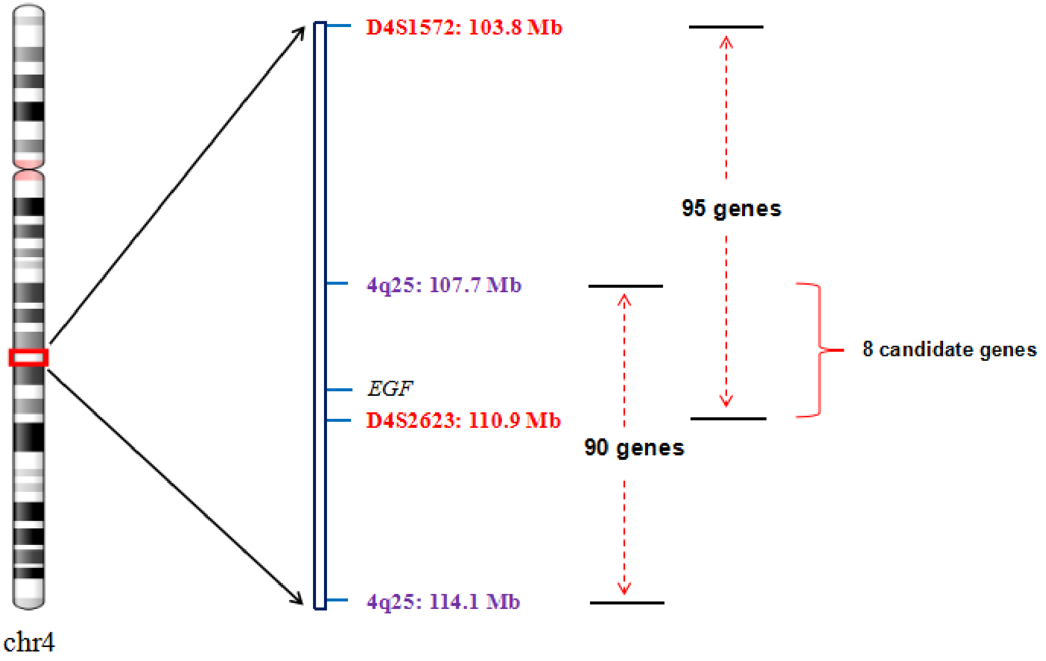

Figure S1- candidate genes were selected from genomic region near marker D4S2623 and 4q25.

References

1 Richette, P. & Bardin, T. Gout. *Lancet* **375**, 318-328, doi:10.1016/S0140-6736(09)60883-7 (2010).

2 Riches, P. L., Wright, A. F. & Ralston, S. H. Recent insights into the pathogenesis of hyperuricaemia and gout. *Human molecular genetics* **18**, R177-184, doi:10.1093/hmg/ddp369 (2009).

3 So, A. & Thorens, B. Uric acid transport and disease. *The Journal of clinical investigation* **120**, 1791-1799, doi:10.1172/JCI42344 (2010).

4 Ford, E. S., Li, C., Cook, S. & Choi, H. K. Serum concentrations of uric acid and the metabolic syndrome among US children and adolescents. *Circulation* **115**, 2526-2532, doi:10.1161/CIRCULATIONAHA.106.657627 (2007).

5 Sun, X. *et al.* Common variants related to serum uric acid concentrations are associated with glucose metabolism and insulin secretion in a Chinese population. *PloS one* **10**, e0116714, doi:10.1371/journal.pone.0116714 (2015).

6 Babio, N. *et al.* Associations between serum uric acid concentrations and metabolic syndrome and its components in the PREDIMED study. *Nutrition, metabolism, and cardiovascular diseases : NMCD* **25**, 173-180, doi:10.1016/j.numecd.2014.10.006 (2015).

7 Choi, Y. J. *et al.* Uric acid induces fat accumulation via generation of endoplasmic reticulum stress and SREBP-1c activation in hepatocytes. *Laboratory investigation; a journal of technical methods and pathology* **94**, 1114-1125, doi:10.1038/labinvest.2014.98 (2014).

8 Ferre, P. & Foufelle, F. Hepatic steatosis: a role for de novo lipogenesis and the transcription factor SREBP-1c. *Diabetes, obesity & metabolism* **12 Suppl 2**, 83-92, doi:10.1111/j.1463-1326.2010.01275.x (2010).

9 Lanaspa, M. A. *et al.* Uric acid induces hepatic steatosis by generation of mitochondrial oxidative stress: potential role in fructose-dependent and -independent fatty liver. *The Journal of biological chemistry* **287**, 40732-40744, doi:10.1074/jbc.M112.399899 (2012).

10 Xie, Y. *et al.* Serum uric acid and non-alcoholic fatty liver disease in non-diabetic Chinese men. *PloS one* **8**, e67152, doi:10.1371/journal.pone.0067152 (2013).

11 Hardwick, R. N., Fisher, C. D., Canet, M. J., Scheffer, G. L. & Cherrington, N. J. Variations in ATP-binding cassette transporter regulation during the progression of human nonalcoholic fatty liver disease. *Drug metabolism and disposition: the biological fate of chemicals* **39**, 2395-2402, doi:10.1124/dmd.111.041012 (2011).

12 Devos, A. *et al.* Genetic polymorphism of CYP2U1, a cytochrome P450 involved in fatty acids hydroxylation. *Prostaglandins, leukotrienes, and essential fatty acids* **83**, 105-110, doi:10.1016/j.plefa.2010.06.005 (2010).

13 Chuang, S. S. *et al.* CYP2U1, a novel human thymus- and brain-specific cytochrome P450, catalyzes omega- and (omega-1)-hydroxylation of fatty acids. *The Journal of biological chemistry* **279**, 6305-6314, doi:10.1074/jbc.M311830200 (2004).

14 Morcillo, S. *et al.* ELOVL6 genetic variation is related to insulin sensitivity: a new candidate gene in energy metabolism. *PloS one* **6**, e21198, doi:10.1371/journal.pone.0021198 (2011).

15 Murakami, M. *et al.* Recent progress in phospholipase A(2) research: from cells to animals to humans. *Progress in lipid research* **50**, 152-192, doi:10.1016/j.plipres.2010.12.001 (2011).

16 Murakami, M., Taketomi, Y., Girard, C., Yamamoto, K. & Lambeau, G. Emerging roles of secreted phospholipase A2 enzymes: Lessons from transgenic and knockout mice. *Biochimie* **92**,

561-582, doi:10.1016/j.biochi.2010.03.015 (2010).
17 Hailemariam, T. K. *et al.* Sphingomyelin synthase 2 deficiency attenuates NFkappaB activation. *Arteriosclerosis, thrombosis, and vascular biology* **28**, 1519-1526, doi:10.1161/ATVBAHA.108.168682 (2008).
18 Li, Y. *et al.* Sphingomyelin synthase 2 activity and liver steatosis: an effect of ceramide-mediated peroxisome proliferator-activated receptor gamma2 suppression. *Arteriosclerosis, thrombosis, and vascular biology* **33**, 1513-1520, doi:10.1161/ATVBAHA.113.301498 (2013).
19 Modan, M., Halkin, H., Karasik, A. & Lusky, A. Elevated serum uric acid--a facet of hyperinsulinaemia. *Diabetologia* **30**, 713-718 (1987).
20 Quinones Galvan, A. *et al.* Effect of insulin on uric acid excretion in humans. *The American journal of physiology* **268**, E1-5 (1995).
21 Snider, K. E. *et al.* Genotype and phenotype correlations in 417 children with congenital hyperinsulinism. *The Journal of clinical endocrinology and metabolism* **98**, E355-363, doi:10.1210/jc.2012-2169 (2013).
22 Kapoor, R. R. *et al.* Clinical and molecular characterisation of 300 patients with congenital hyperinsulinism. *European journal of endocrinology / European Federation of Endocrine Societies* **168**, 557-564, doi:10.1530/EJE-12-0673 (2013).
23 Cronstein, B. N. & Terkeltaub, R. The inflammatory process of gout and its treatment. *Arthritis research & therapy* **8 Suppl 1**, S3, doi:10.1186/ar1908 (2006).
24 Busso, N. & So, A. Mechanisms of inflammation in gout. *Arthritis research & therapy* **12**, 206, doi:10.1186/ar2952 (2010).
25 Kasza, A. IL-1 and EGF regulate expression of genes important in inflammation and cancer. *Cytokine* **62**, 22-33, doi:10.1016/j.cyto.2013.02.007 (2013).
26 Hou, Y. *et al.* N-acetylcysteine reduces inflammation in the small intestine by regulating redox, EGF and TLR4 signaling. *Amino acids* **45**, 513-522, doi:10.1007/s00726-012-1295-x (2013).
27 Steinke, F. C. & Xue, H. H. From inception to output, Tcf1 and Lef1 safeguard development of T cells and innate immune cells. *Immunologic research* **59**, 45-55, doi:10.1007/s12026-014-8545-9 (2014).
28 Gornik, O. & Lauc, G. Glycosylation of serum proteins in inflammatory diseases. *Disease markers* **25**, 267-278 (2008).
29 Yucel-Lindberg, T. & Brunius, G. Epidermal growth factor synergistically enhances interleukin-8 production in human gingival fibroblasts stimulated with interleukin-1beta. Archives of oral biology **51**, 892-898, doi:10.1016/j.archoralbio.2006.03.004 (2006).
30 Yu, S. et al. The TCF-1 and LEF-1 transcription factors have cooperative and opposing roles in T cell development and malignancy. Immunity **37**, 813-826, doi:10.1016/j.immuni.2012.08.009 (2012).
31 Steinke, F. C. et al. TCF-1 and LEF-1 act upstream of Th-POK to promote the CD4(+) T cell fate and interact with Runx3 to silence Cd4 in CD8(+) T cells. Nature immunology 15, 646-656, doi:10.1038/ni.2897 (2014).
